# POLRMT over‐expression is linked to WNT/beta‐catenin signaling, immune infiltration, and unfavorable outcomes in lung adenocarcinoma patients

**DOI:** 10.1002/cam4.6174

**Published:** 2023-06-07

**Authors:** Yongkang Huang, Yajuan Qian, Yufei Xing, Yongjian Pei, Beilei Zhang, Ting Li, Xue Pan, Anyuan Zhong, Juan Du, Tong Zhou, Minhua Shi

**Affiliations:** ^1^ Department of Respiratory and Critical Care Medicine The Second Affiliated Hospital of Soochow University Suzhou Jiangsu China; ^2^ Department of Respiratory and Critical Care Medicine The First Affiliated Hospital of Soochow University Suzhou Jiangsu China

**Keywords:** immune infiltration, lung adenocarcinoma, outcome, pathway, POLRMT, Wnt signal

## Abstract

**Background:**

Mitochondrial RNA polymerase (POLRMT) is essential for the expression of mitochondrial genes. In recent studies, POLRMT expression promoted non‐small cell cancer cell proliferation in cell lines and xenografts. The present study investigated the impact of POLRMT expression and function on lung adenocarcinoma (LUAD) patients.

**Method:**

Multi‐omics data (genomics, transcriptomics, and proteomics) from publicly available databases were used to assess the role of POLRMT expression and function in LUAD. These findings were further verified using cancer tissues from clinical samples.

**Results:**

POLRMT was over‐expressed in LUADs, with mutation frequencies ranging from 1.30% to 5.71%. Over‐expression of POLRMT was associated with an abnormal clinicopathological condition resulting in a decreased lifespan. Furthermore, gene sets enrich analysis revealed that POLRMT expression was linked to WNT/beta‐catenin signaling; the expression of downstream target genes was positively correlated with POLRMT expression. Also, POLRMT expression was positively correlated with immunosuppressive genes, thereby affecting immune infiltration.

**Conclusion:**

POLRMT is over‐expressed in LUAD, thereby impacting patient survival. It is also involved in WNT/beta‐catenin signaling and may affect tumor infiltration.

## 1 BACKGROUND

In recent decades, lung cancer has remained one of the most prevalent form of cancer with 2.2 million cases globally in 2020,^1^ affecting families and creating a huge societal burden. Lung adenocarcinoma (LUAD) is the most frequent subtype seen in ~40% of lung cancers.^2^ Chemotherapy is the conventional treatment choice, with targeted molecular therapy and immune checkpoint inhibitor therapy being the more personalized treatment options. However, neither treatment option is useful in mitigating drug resistance or tumor progression. Therefore, early diagnosis and exploring other treatment options are vital.

Mitochondrial RNA polymerase (POLRMT) is a highly conserved, nuclear‐encoded RNA polymerase required for the expression of mitochondrial genes, which encode subunits of oxidative phosphorylation complexes.^3^, ^4^ Recruited by TFAM and assisted by TFB2M, POLRMT initiates the transcription of mtDNA. Mutations in the gene encoding POLRMT directly impact transcription levels, thereby causing dysfunction in key mitochondrial processes such as oxidative phosphorylation, eventually leading to the development of malignant tumors. A growing body of evidence indicates that POLRMT expression is associated with various cancers, including skin squamous cell carcinoma,^5^ osteosarcoma,^6^ acute myeloid leukemia,^7^, ^8^ and breast cancer.^9^ Our recent study also revealed that POLRMT expression promoted non‐small cell lung cancer (NSCLC) proliferation in cell lines and xenografts^10^; however, the study did not use tissues from cancer patients to examine the role of POLRMT in tumor formation and shed less light on underlying mechanism.

## 2 METHODS

### 2.1 Ethical approval and tissue sample collection

In total, 117 NSCLC tissues and adjacent normal tissues were collected from patients at the Department of Thoracic Surgery, Second Affiliated Hospital of Soochow University between 2016 and 2017 after obtaining written consent. All tissue samples were stored at −80°C. All patients in the study were pathologically diagnosed with lung cancer after surgery and did not exhibit any other severe health complications (e.g., systemic infections or organ failure). The clinical data of patients were collected, which included age, gender, smoking history, histopathological grade, carcinoembryonic antigen, and TNM classification, the overall survival (OS). The survival data were ultimately collected by February 2022.This study was approved by the ethics committee of the Second Affiliated Hospital of Soochow University after review.

### 2.2 DNA mutation, methylation, and expression of POLRMT

We investigated the somatic mutation frequency of POLRMT in LUAD data from the cBioPortal database (https://www.cbioportal.org/), giving preference to the dataset with the larger sample size if there were overlapping samples. The promoter methylation level of LUAD was assessed based on The Cancer Genome Atlas Lung Adenocarcinoma Project (TCGA‐LUAD) using the UALCAN website^11^ (https://ualcan.path.uab.edu/). For mRNA expression analysis, RNA sequence data on LUAD were obtained from TCGA‐LUAD using the GDC data portal (https://portal.gdc.cancer.gov/), and transcripts per kilobase million were extracted for POLRMT expression analysis. Gene chip data were acquired from the Gene Expression Omnibus (GEO) database (https://www.ncbi.nlm.nih.gov/geo/) using the search term “lung adenocarcinoma” with organism, entry type, and study type restricted to *Homo sapiens*, serial, and expression profiling by array, respectively. We preferred datasets that contained both tumor and normal samples with comparable sample sizes, and ultimately chose two datasets (GSE46539 and GSE10072) to investigate POLRMT expression. Protein expression was evaluated using Clinical Proteomic Tumor Analysis Consortium (CPTAC) samples analyzed via the UALCAN website.^11^

### 2.3 Gene set enrichment analysis and correlation analysis

Tumor samples from TCGA‐LUAD were divided into POLRMT‐high and POLRMT‐low groups based on median POLRMT expression. The counts of each gene were extracted to calculate log2‐fold change using the edgeR package^12^ in R. Genes were sorted in descending order according to log2‐fold change for further analysis. Gene set enrichment analysis (GSEA) was performed using the cluster profile package^13^ and FGSEA packages^14^ in R, with the ordered genes. Annotated gene sets for GSEA were obtained from The Molecular Signatures Database (MSigDB) (http://www.gsea‐msigdb.org/gsea/msigdb/index.jsp), including hallmark gene sets,^15^ canonical pathways subset (C2), Gene Ontology gene sets (C5), and ImmuneSigDB subset of immunologic signature gene sets (C7). Correlation analysis between POLRMT and genes of interest was conducted using the correlation module in TIMER (https://cistrome.shinyapps.io/timer/), an online tool that can draw expression scatterplots and provide Spearman's rho value and statistical significance for given genes.^16^

### 2.4 Immune cell infiltration analysis

The correlation between the expression of POLRMT and immune cell infiltration was examined using CIBERSORT^17^ in R, as previously described.^18^ FPKM data from TCGA‐LUAD tumor samples was utilized in the analysis. The leukocyte signature matrix (LM22) was obtained from CIBERSORTx (https://cibersortx.stanford.edu) and used as the signature gene file.

### 2.5 Immunohistochemical assays

Of 117 pairs of paraffin‐embedded NSCLC tissues, 113 LUAD were selected for immunohistochemical (IHC) assays. After paraffin detachment and rehydration, epitopes of the tissues were retrieved by microwaving in antigen retrieval buffer. The slides were then washed gently by soaking in phosphate‐buffered saline three times. Next, endogenous peroxidase and non‐specific binding sites were blocked by submerging the slides in 0.3% H_2_O_2_ for 15–40 min and overlaying them with 10% normal serum blocking reagent for 1 h at room temperature. Subsequently, a diluted solution composed of the primary antibody and blocking reagent was applied to the tissues with parafilm for 2 h in a humidified container at room temperature. The tissues were then incubated with biotinylated secondary antibodies in a block with Cell G reagent for 1 h after elution, followed by incubation with 3,3′‐diaminobenzidine tetra‐hydrochloride and 10% Mayer's hematoxylin to counterstain nuclei. Finally, the prepared slides were viewed under a light microscope for protein detection.

### 2.6 Evaluating POLRMT expression

Clinical samples were divided into subgroups based on the expression scores of POLRMT, which were calculated by immunohistochemistry. An empirical value of 4 was set as the cutoff value to distinguish between high‐ and low‐level expressions. The expression score was determined as the product of two factors: the total number of cells stained, which were categorized as 0 (negative), 1 (weak), 2 (moderate), or 3 (strong), and the intensity of staining, which was categorized as 1 (0%–25% positive), 2 (26%–50% positive), 3 (51%–75% positive), or 4 (76%–100% positive).

### 2.7 Statistical analysis

Statistical analyses were performed and visualized in R (version 4.1.1) or SPSS (version 17.0). For continuous and dichotomous variables, we report the mean and standard deviation, frequency, and proportion. The *t*‐test was used for continuous variables while Pearson's chi‐square test or the Mann–Whitney *U* test was used for dichotomous variables. The survival data were analyzed and visualized using Kaplan–Meier curve via survival^19^ and survminer^20^ package, respectively. The log‐rank test was employed to ascertain the differences between groups in the survival analysis. Variables got a *p*‐value of no more than 0.05 on univariate Cox regression analysis were subjected to multivariate Cox regression models when exploring the risk factors for lung cancer survival. A *p*‐value <0.05 indicated statistical significance.

## 3 RESULTS

### 3.1 POLRMT was over‐expressed in LUAD

**FIGURE 1:**
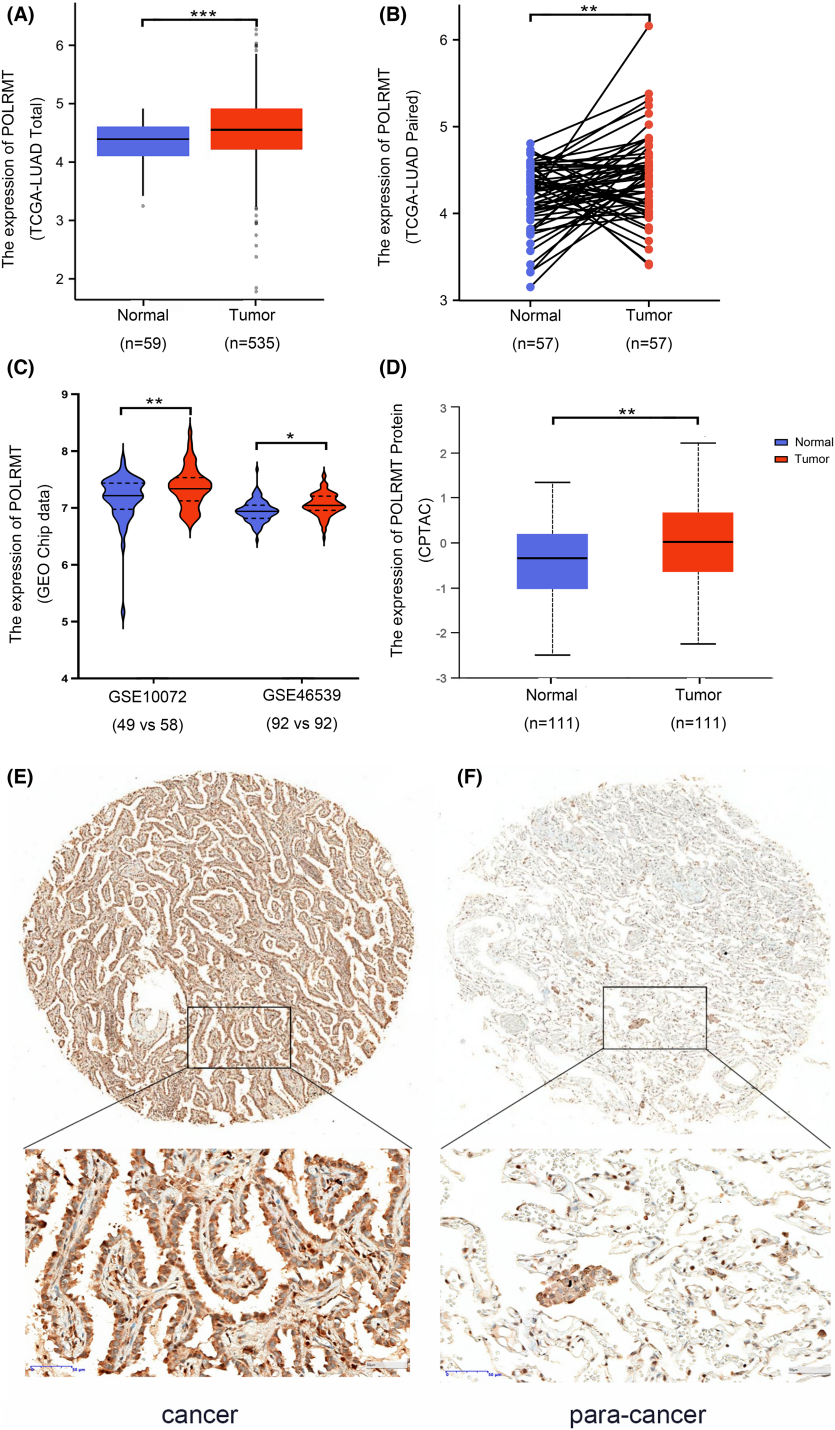
POLRMT was over‐expressed in LUAD. (A–C) POLRMT mRNA expression in TCGA‐LUAD patients [(A), total samples; (B), paired samples) and GEO datasets (C)]. (D–F) POLRMT protein expression in CPTAC LUAD samples (D) and clinical patients (E, F). *p < 0.05; **p < 0.01; ***p < 0.001. CPTAC, Clinical Proteomic Tumor Analysis Consortium; GEO, Gene Expression Omnibus; IHC, immunohistochemical; LUAD, lung adenocarcinoma; POLRMT, mitochondrial RNA polymerase; TCGA, The Cancer Genome Atlas.

We first investigated POLRMT expression using RNA sequence data from TCGA database. POLRMT was found to be significantly over‐expressed in the tumor samples (Figure 1A). Considering the uneven numbers of tumor and normal samples in TCGA‐LUAD, a paired analysis was performed that revealed elevated POLRMT expression (Figure 1B). We confirmed POLRMT over‐expression using array‐based data from the GEO genomics data repository (Figure 1C). Protein expression was consistent with RNA expression in LUAD as seen in CPTAC samples (Figure 1D).

To verify these findings, an IHC assay was performed using surgical samples from clinical patients (*n* = 113). There were 39 cases of strong POLRMT expression in the tumor samples (*n* = 39/113, 34.5%), while there were only 11 cases of strong POLRMT expression in the adjacent normal tissues (*n* = 11/113, 9.7%). The inter‐group difference was found to be significant (*p* < 0.0001).

### 3.2 High‐level POLRMT expression was associated with unfavorable clinicopathological characteristics resulting in decreased survival

To assess the impact of POLRMT expression on patients, we analyzed the differences in clinicopathological characteristics between high‐ and low‐level POLRMT‐expressing subgroups. The subgroup strongly expressing POLRMT had larger tumors, a greater incidence of metastasis, and a worse TNM stage (Table 1), suggesting a clear role for POLRMT in cancer development.

**TABLE 1:** Clinicopathological characteristics between the high and low POLRMT subgroup.

	POLRMT‐high	POLRMT‐low	*p*‐value
Age			0.404
≤65	20	44	
>65	19	30	
Gender			0.112
Male	23	32	
Female	16	42	
Smoke			0.865
No	28	52	
Yes	11	22	
CEA			0.447
Normal	23	49	
Up	16	25	
Histopathological grade			0.099
I	8	7	
II/III	31	67	
T stage			0.002**
T1/T2	28	69	
T3/T4	11	5	
N stage			0.962
N0	26	49	
N1/N2/N3	13	25	
M stage			<0.001***
M0	28	70	
M1	11	4	
TNM classification			0.0448*
I/II	23	57	
III/IV	16	17	

Abbreviations: CEA, carcinoembryonic antigen; POLRMT, Mitochondrial RNA polymerase.

* *p* < 0.05

** *p* < 0.01

*** *p* < 0.001.

To assess whether POLRMT expression affected the resulting outcome of LUAD, we investigated POLRMT expression in patients based on OS and progression‐free survival (PFS) using KM Plotter.^21^ Higher‐level POLRMT expression was found to be associated with shortened OS and PFS (Figure 3A,B).

**FIGURE 2:**
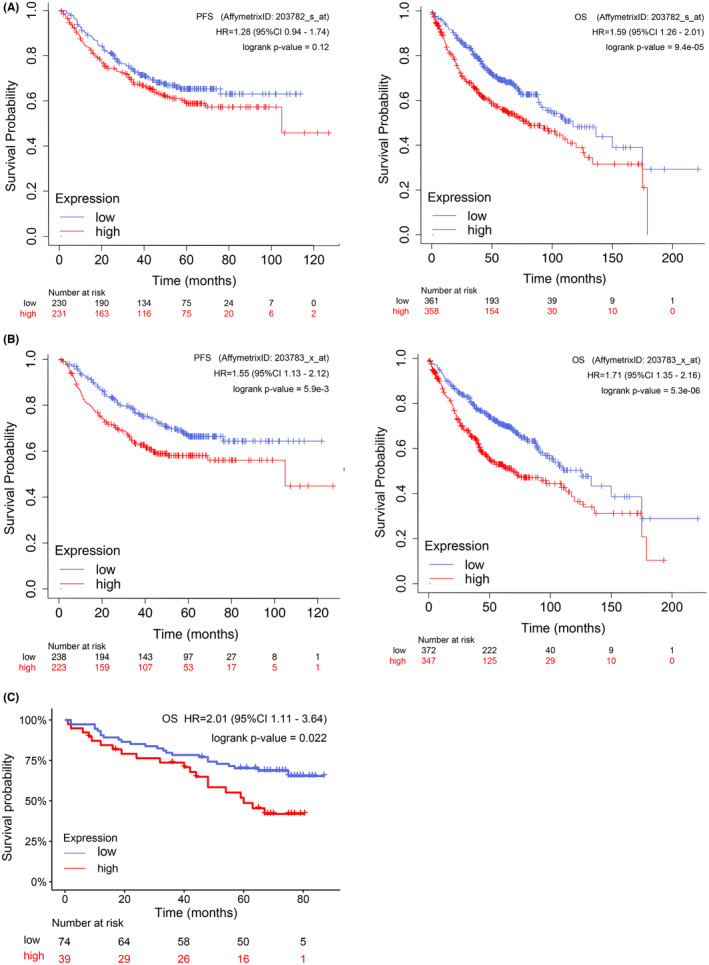
High‐level POLRMT expression was associated with decreased survival. (A, B) High‐level POLRMT expression mean shortened OS and PFS using KM Plotter (A, B). (C) High‐level POLRMT expression mean shortened OS in clinical patients. OS, overall survival; PFS, progression‐free surviva; POLRMT, mitochondrial RNA polymerase.

Further, 113 patients were examined to determine the relationship between POLRMT and OS. Consistent with our findings using data from online databases, the Kaplan–Meier curve showed that high‐level expression of POLRMT was associated with a poorer survival outcome (hazard ratio = 2.01, *p* = 0.022) (Figure 2C). As TNM stage also predicts patients outcome, we further conducted Cox regression hazard model to obtain an adjusted hazard ratio of POLRMT equal to 1.89 (*p* = 0.037) (Table S1). Moreover, a ROC curve indicated POLRMT expression could be used as a predictor for survival in LUAD, with area under the curve equal to 0.63 (95% confidence interval: 0.52–0.73) (Figure S1).

### 3.3 POLRMT mutation frequency and DNA methylation in LUAD

**FIGURE 3:**
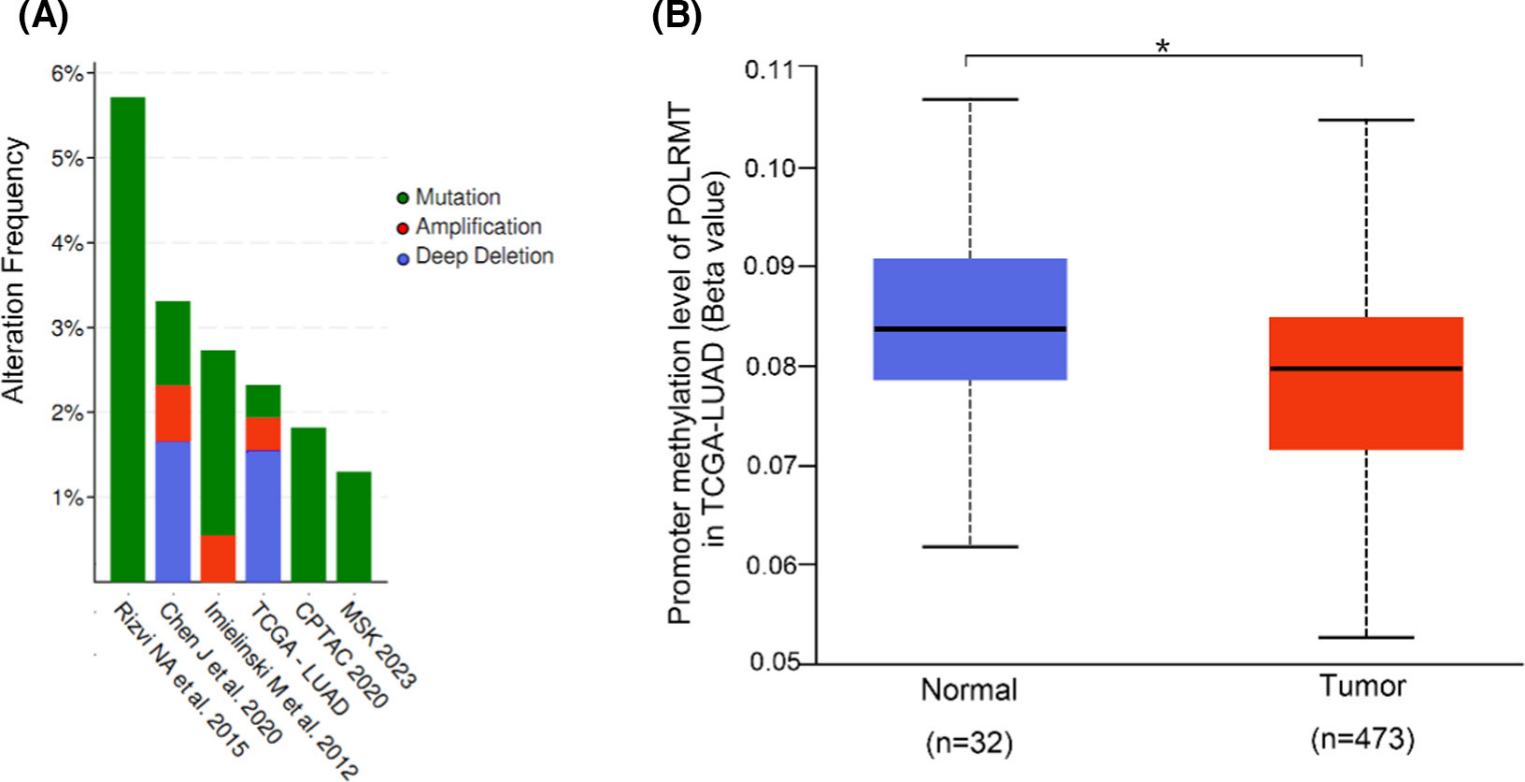
POLRMT mutation frequency and DNA methylation in LUAD. (A) Alteration frequency of POLRMT in LUAD. (B) Promoter methylation level of POLRMT in TCGA‐LUAD. LUAD, lung adenocarcinoma; POLRMT, mitochondrial RNA polymerase; TCGA, The Cancer Genome Atlas. *p < 0.05.

After filtering out datasets with overlapping samples and those without mutation data, 6 out of 14 LUAD datasets in cBioPortal were eligible for exploring POLRMT DNA mutations. We observed frequencies of 1.30%–5.71% in the available datasets, including Rizvi et al.^22^, Chen et al.^23^, Imielinski et al.^24^, TCGA‐LUAD, CPTAC^25^ with deletions and amplifications as the most common mutation types (Figure 3A). We also found that the methylation state of POLRMT was lower in tumor samples than in normal samples (*p* = 0.023) (Figure 3B); this was negatively co‐related to the POLRMT expression level in TCGA‐LUAD (Pearson rho = −0.15, *p* < 0.001) and CPTAC datasets (Pearson rho = −0.07, *p* = 0.514) (Figure S2).

### 3.4 POLRMT expression is associated with WNT/beta‐catenin signaling

**FIGURE 4:**
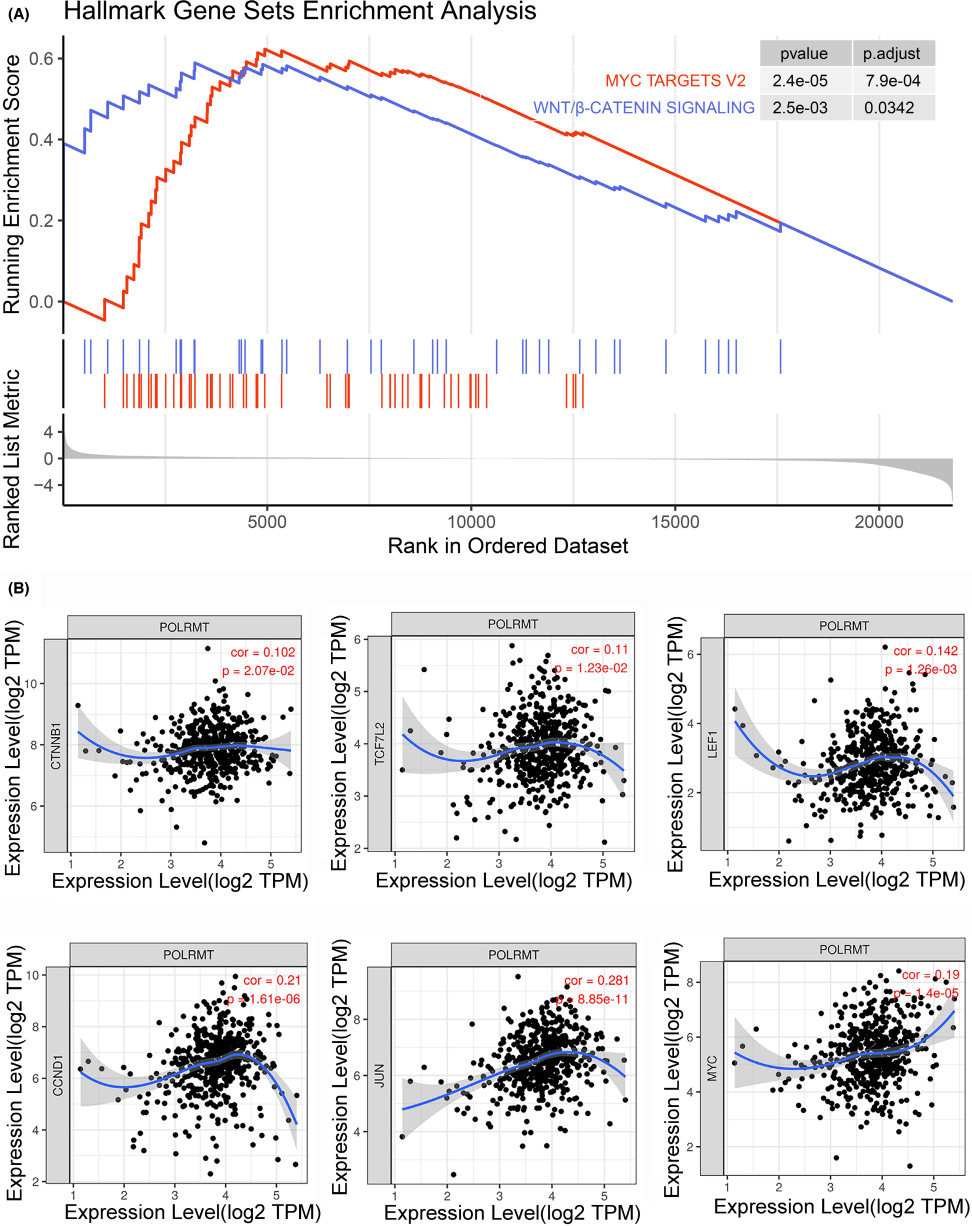
POLRMT expression is associated with WNT/beta‐catenin signaling. (A) High POLRMT expression levels activated MYC Targets and WNT/beta‐catenin signaling pathway. (B) Positive correlation between POLRMT and downstream target genes of WNT/beta‐catenin. POLRMT, mitochondrial RNA polymerase.

As the POLRMT expression level was associated with an unfavorable clinical outcome in patients and promoted NSCLC proliferation in cell lines and xenografts as shown in our previous study,^10^ tumor samples from TCGA were divided into two subgroups (high‐level and low‐level POLRMT expression) to dissect the underlying mechanism. GSEA of hallmark gene sets revealed that high‐POLRMT expression levels activated MYC Targets and WNT/beta‐catenin signaling pathway (Figure 4A). Given that MYC was also a downstream target gene of the WNT/beta‐catenin signaling pathway,^26^ we further assessed the correlation between POLRMT expression and downstream target genes of WNT/beta‐catenin, including beta‐catenin, TCF, c‐myc, c‐jun, and cyclin D1. These downstream target genes were found to be positively correlated with POLRMT expression (Figure 4B), indicating that POLRMT over‐expression is linked to WNT/beta‐catenin signaling.

Moreover, GSEA of vital biological process‐related gene sets like canonical pathways and gene ontology biological process subsets revealed that metabolism and synthesis of steroid hormones and three primary nutrients (including amino acids, lipids, and glucose) were upregulated. In addition, gene sets responsible for body immunity, signal transduction, and material transport were upregulated, showing a clear impact of POLMRT on these genes (Table 2).

**TABLE 2:** The top 30 biological process‐related gene sets ordered by normalized enrichment score (NES).

ID	Category	NES	*q*‐value
Glucocorticoid_Metabolic_Process	GO biological process	2.354	0.0016
C21_Steroid_Hormone_Metabolic_Process	GO biological process	2.269	0.0016
Metabolism_of_Steroid_Hormones	Reactome gene sets	2.204	0.0049
Killing_By_Host_of_Symbiont_Cells	GO biological process	2.190	0.0113
Steroid_Hormone_Biosynthetic_Process	GO biological process	2.175	0.0146
Glucocorticoid_Biosynthetic_Process	GO biological process	2.173	0.0017
Organ_or_Tissue_Specific_Immune_Response	GO biological process	2.138	0.0249
Antimicrobial_Peptides	Reactome gene sets	2.071	0.0072
Classical_Pathway_of_Steroidogenesis_With_Glucocorticoid_And_Mineralocorticoid_Metabolism	WikiPathways gene sets	2.071	0.0072
Response_To_Food	GO biological process	2.062	0.0348
Hippocampus_Development	GO biological process	2.052	0.0103
Hormone_Biosynthetic_Process	GO biological process	2.048	0.0106
Corticotropinreleasing_Hormone_Signaling_Pathway	WikiPathways gene sets	2.040	0.0017
Kinesins	Reactome gene sets	2.028	0.0072
Steroid_Hormone_Biosynthesis	KEGG pathway	2.018	0.0188
Neuropeptide_Signaling_Pathway	GO biological process	2.012	0.0164
Defense_Response_To_Gram_Negative_Bacterium	GO biological process	2.011	0.0164
Monoamine_Transport	GO biological process	1.996	0.0146
Metabolism_of_Fat_Soluble_Vitamins	Reactome gene sets	1.975	0.0198
Limbic_System_Development	GO biological process	1.962	0.0103
Recruitment_of_Numa_To_Mitotic_Centrosomes	Reactome gene sets	1.944	0.0067
Sleep_Regulation	WikiPathways gene sets	1.939	0.0413
Alternative_Pathway_of_Fetal_Androgen_Synthesis	WikiPathways gene sets	1.931	0.0376
HDL_Remodeling	Reactome gene sets	1.919	0.0116
Recycling_Pathway_of_L1	Reactome gene sets	1.918	0.0412
Chylomicron_Assembly	Reactome gene sets	1.908	0.0143
Transport_of_Bile_Salts_And_Organic_Acids_Metal_Ions_And_Amine_Compounds	Reactome gene sets	1.896	0.0072
Acute_Inflammatory_Response	GO biological process	1.876	0.0169
Adipocytokine_Signaling_Pathway	KEGG pathway	1.872	0.0259
Cellular_Hormone_Metabolic_Process	GO biological process	1.870	0.0042

### 3.5 POLRMT could affect tumor immune infiltration

**FIGURE 5:**
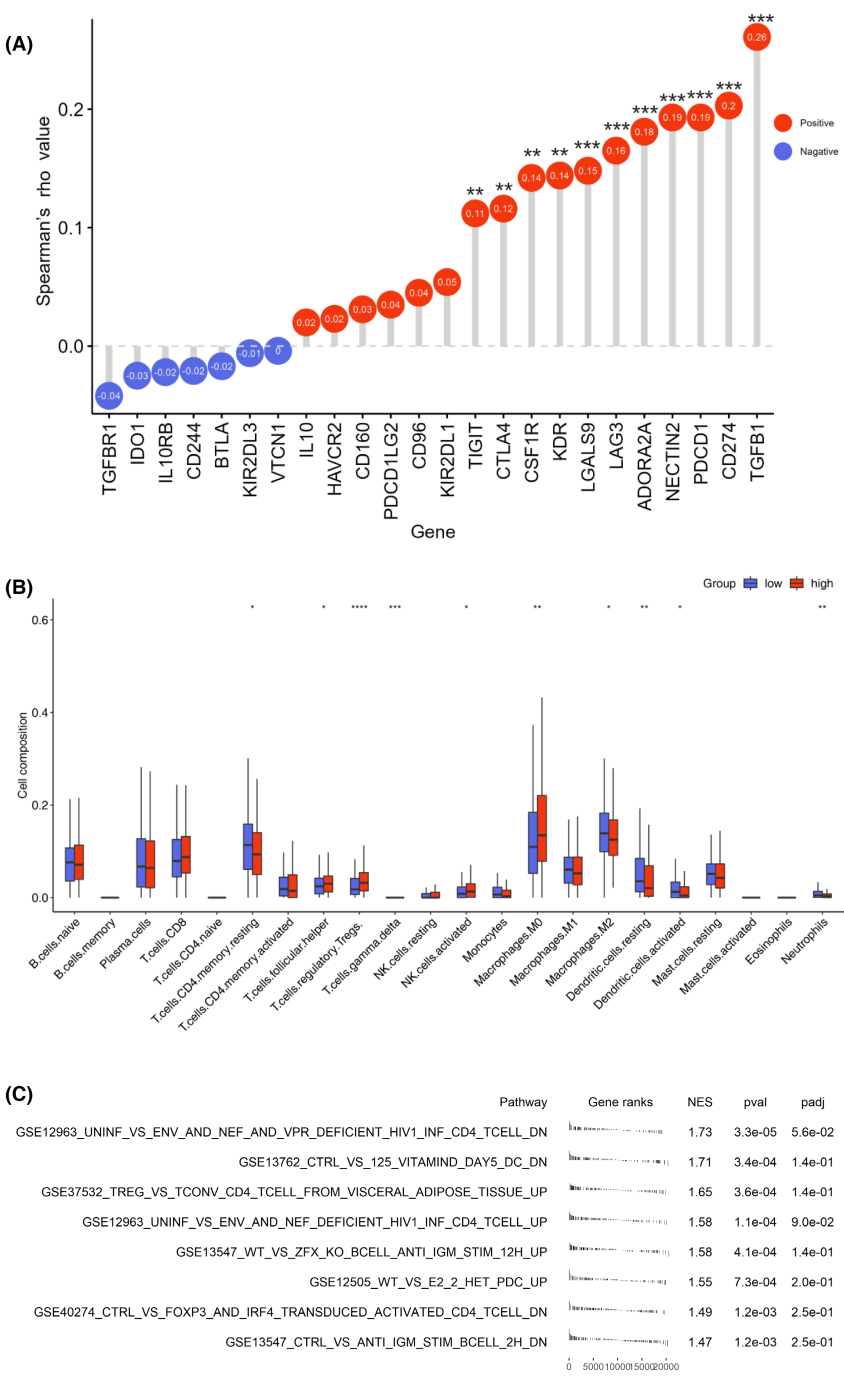
POLRMT could affect tumor immune infiltration. (A) Correlation of POLRMT expression with commonly accepted immunosuppressive genes. (B) POLMRT expression and tumor immune infiltration. (C) GSEA of ImmuneSigDB subset of immunologic signature gene sets. *p < 0.05; **p < 0.01; ***p < 0.001. GSEA, gene set enrich analysis; POLRMT, mitochondrial RNA polymerase.

Tumor development is linked to body immunity, and our GSEA results showed the correlation of POLRMT with body immunity. To assess this impact in detail, we first studied the correlation of POLRMT expression with commonly accepted immunosuppressive genes. As shown in Figure 5A, POLRMT expression was positively correlated with multiple genes, including *TIGIT*, *CTLA4*, *CSF1R*, *LAG3*, and *PDCD1* (Figure 5A). Furthermore, we analyzed the correlation between POLRMT expression and widely recognized markers of immune cells, including B cells, T cells (Th1, Th2, follicular helper T cells, Th17, regulatory T [Treg] cells, and CD8^+^ T cells), natural killer (NK) cells, monocytes, neutrophils, dendritic cells (DCs), and macrophages using TIMER2.^27^ Our results clearly indicated that POLRMT expression was relevant to markers of these cells except for CD8^+^ T cells. Treg cell markers were positively associated with POLRMT (Table 3). These findings suggested that high expression of POLRMT might indicate a relatively immunosuppressive environment.

**TABLE 3:** Correlation between POLRMT and gene markers of immune cells.

Cell	Gene	None		Tumor purity‐adjusted		Age adjusted	
		Rho	*p*‐value	Rho	*p*‐value	Rho	*p*‐value
B cell	CD19	0.114	**	0.112	*	0.122	**
	CD79A	0.043	0.331	0.031	0.491	0.053	0.241
Th1	TBX21	0.099	*	0.088	0.0504	0.112	*
	STAT4	0.085	0.053	0.059	0.189	0.107	*
	STAT1	0.097	*	0.074	0.0995	0.114	*
	IFNG	0.042	0.347	0.029	0.520	0.044	0.334
	TNF	0.093	*	0.089	*	0.121	**
	IL12A	0.147	***	0.128	**	0.158	***
	IL12B	−0.007	0.867	−0.013	0.765	0.002	0.972
Th2	GATA3	0.161	***	0.161	***	0.185	***
	STAT6	0.127	**	0.129	**	0.144	**
	STAT5A	0.240	***	0.252	***	0.267	***
	IL13	0.083	0.059	0.082	0.070	0.079	0.083
	BCL6	0.196	***	0.196	***	0.200	***
	IL21	0.085	0.053	0.073	0.104	0.075	0.098
Th17	STAT3	0.098	*	0.101	*	0.118	**
	IL17A	0.019	0.660	0.012	0.796	0.009	0.836
Treg	FOXP3	0.177	***	0.173	***	0.198	***
	CCR8	0.096	*	0.083	0.064	0.117	*
	STAT5B	0.267	***	0.273	***	0.269	***
	TGFB1	0.261	***	0.262	***	0.282	***
CD8+ T cell	CD8A	0.037	0.396	0.023	0.613	0.041	0.364
	CD8B	−0.004	0.920	−0.026	0.561	−0.006	0.891
Monocyte	CD86	0.017	0.705	0.003	0.952	0.039	0.388
	CD115	0.142	**	0.151	***	0.17	***
M1 macrophage	INOS	0.066	0.133	0.05	0.264	0.085	0.0618
	IRF5	0.258	***	0.243	***	0.264	***
	PTGS2	0.089	*	0.073	0.108	0.072	0.112
M2 macrophage	CD163	0.134	**	0.144	**	0.16	***
	Z39Ig	0.006	0.890	0.001	0.990	0.027	0.559
	MS4A4A	−0.015	0.733	−0.021	0.647	0.007	0.870
Dendritic cell	HLA‐DPB1	0.008	0.799	−0.002	0.783	0.043	0.568
	HLA‐DQB1	0.064	***	0.062	***	0.099	***
	HLA‐DRA	−0.087	0.141	−0.109	0.204	−0.056	0.0666
	HLA‐DPA1	−0.03	0.221	−0.041	0.438	0.003	0.136
	CD1C	−0.039	0.885	−0.046	0.948	−0.02	0.619
	NRP1	0.154	0.358	0.154	0.619	0.15	0.487
	ITGAX	0.266	0.322	0.28	0.516	0.292	0.341
NK cell	KIR2DL1	0.054	0.684	0.035	0.898	0.068	0.532
	KIR2DL3	−0.006	0.253	−0.003	0.234	−0.023	0.192
	KIR2DL4	0.041	0.616	0.022	0.481	0.032	0.836
	KIR2DS4	0.044	*	0.029	*	0.043	*
	KIR3DL1	0.018	0.125	0.006	0.060	0.028	0.236
	KIR3DL2	0.050	0.635	0.054	0.892	0.059	0.532
	KIR3DL3	0.022	0.848	0.032	0.962	0.009	0.351
	XCL1	−0.105	0.149	−0.111	0.171	−0.094	*
	XCL2	−0.068	*	−0.085	*	−0.054	0.217
	NCR1	0.021	0.499	0.006	0.363	0.028	0.952
Neutrophils	CEACAM8	−0.011	0.372	−0.012	0.304	0.026	0.659
	ITGAM	0.187	***	0.193	***	0.223	***
	CCR7	0.065	***	0.057	***	0.084	***

Abbreviation: POLRMT, mitochondrial RNA polymerase.

* *p* < 0.05

** *p* < 0.01

*** *p* < 0.001.

To extrapolate specific links between POLMRT and tumor immune infiltration, we investigated the tumor immune cell composition in TCGA‐LUAD sequence data using Cibersort. In samples with high‐level POLMT expression, CD4 memory resting T cells, M2 macrophages, resting DCs, activated DCs, and neutrophils showed less infiltration while Treg cells, follicular helper T cells (Tfh), activated NK cells, and M0 macrophages showed more infiltration (Figure 5B).

Furthermore, GSEA was performed using the ImmuneSigDB subset of immunologic signature gene sets generated through manual curation of published studies in human immunology encoding chemical and genetic perturbations of the immune system. Multiple gene sets were significant, involving CD4 T cells (*n* = 4), DCs (*n* = 2), and B lymphocytes (*n* = 2) (Figure 5C).

## 4 DISCUSSION

POLRMT, an RNA transcriptional polymerase indispensable for mitochondrial homeostasis, warrants the attention of tumor researchers. We previously assessed the biological impact of POLRMT in NSCLC.^10^ In this study, we evaluated the impact of POLRMT on the survival outcomes of patients and its possible underlying mechanisms.

First, we studied the expression of POLRMT in LUAD using publicly available data from omics databases and found that POLRMT was over‐expressed at both the RNA and protein levels. And DNA methylation of POLRMT was reduced in tumor tissues as compared to normal tissues. Significantly, we verified the results using IHC in clinical samples collected in our hospital. We further demonstrated that strong expression of POLRMT was linked to unfavorable clinicopathological outcomes (e.g., decreased lifespan), implying that it could be used as a biomarker for early diagnosis and a potential target for drug development. There are putative drugs in the pipeline that target POLRMT and they have already shown promising antitumor effects in mice without causing oxidative phosphorylation dysfunction or toxic effects in normal tissues.^28^

Besides the role of POLRMT in NSCLC, recently published studies have revealed the protumoral role of POLRMT in skin squamous cell carcinoma, osteosarcoma, acute myeloid leukemia, and breast cancer. Considering the role of POLRMT in mtDNA transcription, these studies assessed the transcripts of mtDNA genes (e.g., mitochondrial mRNA levels, subunits of respiratory chain complexes, and S6 phosphorylation), and found that the expression of these genes decreased with decreasing POLRMT expression.^5^, ^6^, ^7^, ^10^ However, these studies did not assess whether POLRMT was involved in any biological pathway linked to cancer development. Through GSEA, we found that high‐level expression of POLRMT was linked to MYC targets and Wnt/beta‐catenin signaling, both of which had a role in lung cancer cell processes and proliferation.^29^, ^30^ Consistently, in our GSEA of MSigDB curated gene sets (C2 collection), mitosis was found to be upregulated in the high‐level POLRMT‐expressing subgroup.

Notably, our further analysis revealed a positive correlation between POLRMT and beta‐catenin (essential for in Wnt/beta‐catenin signaling pathway), TCF and LEF (final effectors of the Wnt Cascade^31^), other important downstream target genes (e.g., c‐myc, c‐jun, and cyclin D1). This clearly indicated that POLRMT expression was relevant to Wnt/beta‐catenin signaling pathway.

Interestingly, studies have shown that aberrant activation of WNT signaling facilitates an immune escape and impedes antitumor immune responses. Hence it is regarded as a potential target for cancer therapy.^32^

Furthermore, we dissected the relationship between POLRMT expression and tumor immune infiltration. As residents of the stroma in the tumor microenvironment, immunocytes play important roles in cancer development and the treatment response in patients. Data indicate that lymphocyte‐rich classical Hodgkin's lymphoma featuring Reed‐Sternberg cells has an excellent prognosis.^33^, ^34^ However, decreased mature DCs were linked to weakened antigen processing ability.^35^ Treg cells and tumor‐associated macrophages tend to assist immune evasion and induce cancer development.^36^ We found that high‐level expression of POLRMT was associated with upregulated Treg cells, higher numbers of M0 macrophages, and lower numbers of activated DCs. It was also found that POLRMT expression was positively co‐related to several immunosuppressive genes, some of which were famous and valuable targets for immunotherapy (e.g., those encoding PDCD1, CD274, CTLA4, and novel immune‐checkpoint inhibitors, including LAG3 and TIGIT).^37^, ^38^ Therefore, POLRMT is potentially associated with immune impression.

Finally, using clinical samples from patients, we confirmed high‐level POLRMT expression in LUAD and dissected the underlying mechanisms using RNA‐Seq data from TCGA. We found that POLRMT expression was related to multiple gene sets and was linked to immune infiltration, opening promising avenues of investigation in cancer research.

## 5 CONCLUSION

POLRMT is over‐expressed in LUAD and is linked to WNT/beta‐catenin signaling; this potentially affects tumor infiltration, leading to unfavorable health outcomes for patients.

## AUTHOR CONTRIBUTIONS

**Yongkang Huang:** Data curation (lead); formal analysis (equal); methodology (equal); visualization (equal); writing – original draft (equal). **Yajuan Qian:** Investigation (equal); writing – original draft (equal). **Yufei Xing:** Investigation (equal); methodology (equal); writing – review and editing (equal). **Yongjian Pei:** Data curation (equal); validation (equal); writing – review and editing (equal). **Beilei Zhang:** Resources (equal); software (equal). **Ting Li:** Investigation (supporting); validation (equal); visualization (supporting). **Xue Pan:** Software (equal); visualization (equal). **Anyuan Zhong:** Software (equal); writing – review and editing (equal). **Juan Du:** Data curation (supporting); validation (equal); writing – review and editing (equal). **Tong Zhou:** Conceptualization (equal); funding acquisition (equal); project administration (equal); supervision (equal). **Minhua Shi:** Conceptualization (equal); funding acquisition (equal); project administration (equal); supervision (equal).

## ETHICS STATEMENT

This study was approved by the Ethics Committee of the Second Affiliated Hospital of Soochow University.

## Supporting information


Figure S1.
Figure S2.

Table S1.

## Data Availability

The datasets used in the study were from online repositories, the names of which and accession number(s) can be found in the article. Datasets about the clinical patients could be obtained from corresponding authors on reasonable request.
