# Supplementary material for: POLRMT over‐expression is linked to WNT/beta‐catenin signaling, immune infiltration, and unfavorable outcomes in lung adenocarcinoma patients
Source: Cancer Med. 2023 Jun 7;12(14):15691–703. doi: 10.1002/cam4.6174 (PMC10417304; doi:10.1002/cam4.6174)

**Figure S1 ROC curve for POLRMT expression**


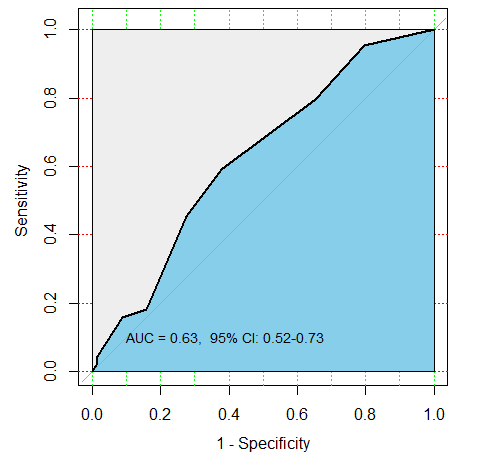


**Figure S2 POLRMT methylation was negatively co-related to the POLRMT expression level in TCGA-LUAD (Pearson rho=-0.15 , p < 0.001) and CPTAC datasets (Pearson rho=-0.07, p = 0.514)**


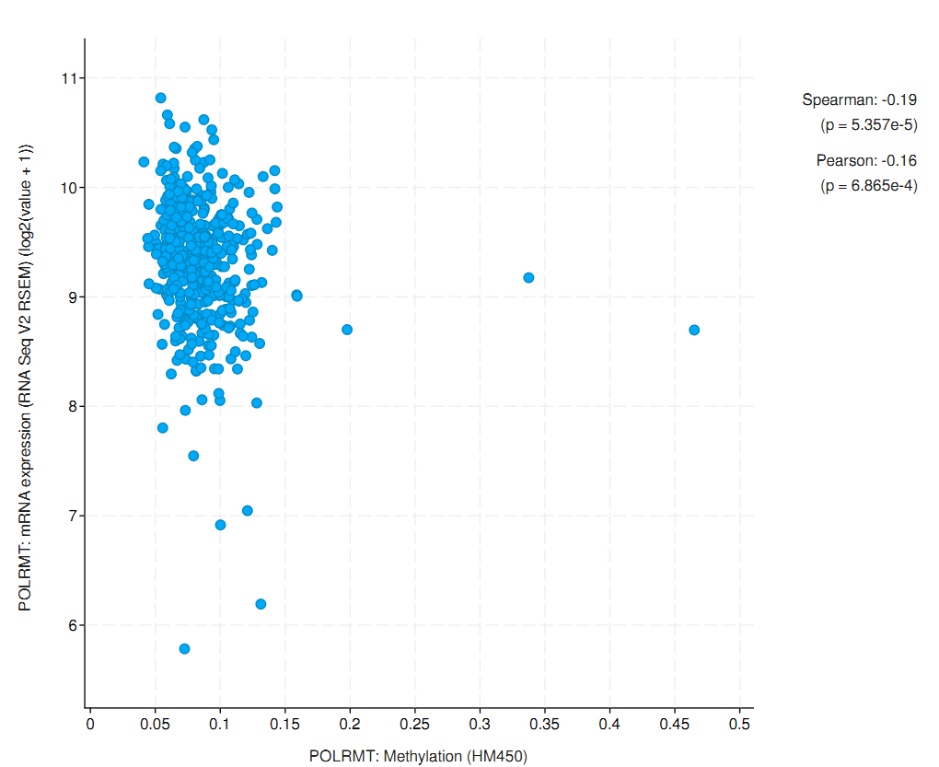


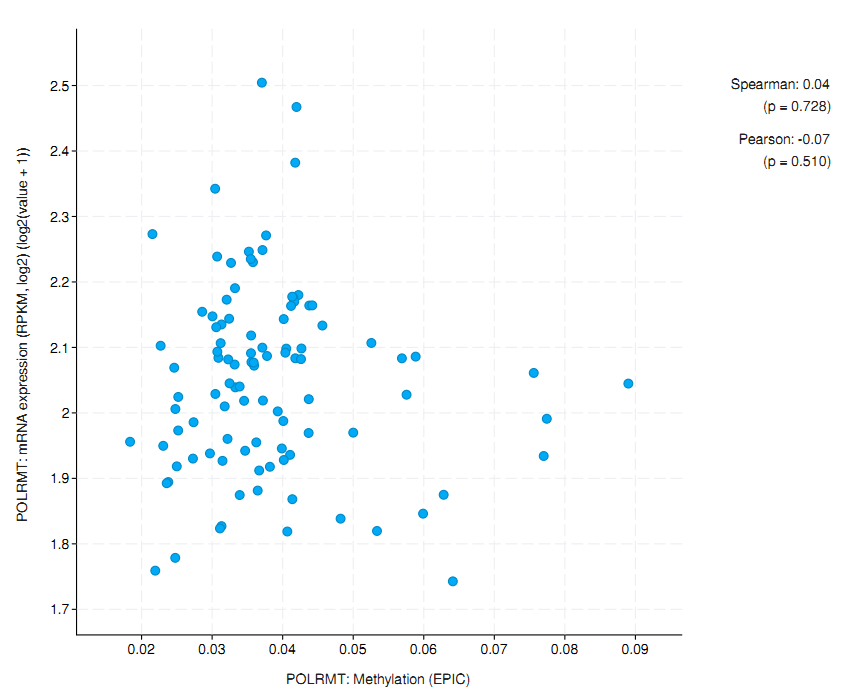

Supplement: Supplementary file 1 — Figure S1. Figure S2. [file CAM4-12-15691-s002.docx]
