# Supplementary material for: POLRMT over‐expression is linked to WNT/beta‐catenin signaling, immune infiltration, and unfavorable outcomes in lung adenocarcinoma patients
Source: Cancer Med. 2023 Jun 7;12(14):15691–703. doi: 10.1002/cam4.6174 (PMC10417304; doi:10.1002/cam4.6174)
Supplement: Supplementary file 2 — Table S1. [file CAM4-12-15691-s001.docx]

| **Table S1 Univariate and multivariate analysis of variables associated with survival** | | | | | | |
| --- | --- | --- | --- | --- | --- | --- |
| Characteristic | HR | 95% CI | p-value | HR adjusted | 95% CI adjusted | p-value adjusted |
| age |  |  |  |  |  |  |
| <65 | reference | |  |  |  |  |
| >65 | 1.20 | 0.66, 2.17 | 0.545 |  |  |  |
| sex |  |  |  |  |  |  |
| female | reference | |  |  |  |  |
| male | 1.26 | 0.70, 2.27 | 0.448 |  |  |  |
| smoke |  |  |  |  |  |  |
| No | reference | |  |  |  |  |
| Yes | 0.76 | 0.38, 1.51 | 0.434 |  |  |  |
| CEA |  |  |  |  |  |  |
| Normal | reference | |  |  |  |  |
| Up | 1.72 | 0.95, 3.12 | 0.072 |  |  |  |
| Histopathological grade |  |  |  |  |  |  |
| 1 | reference | |  |  |  |  |
| 2 / 3 | 0.84 | 0.37, 1.89 | 0.677 |  |  |  |
| TNM stage |  |  |  |  |  |  |
| I/II | reference | |  |  |  |  |
| III/IV | 2.35 | 1.28, 4.30 | 0.006* | 2.24 | 1.22;4.11 | 0.0095* |
| POLRMT Expression |  |  |  |  |  |  |
| low | reference | |  |  |  |  |
| high | 2.01 | 1.11, 3.64 | 0.022* | 1.89 | 1.04;3.45 | 0.037* |
| Abbreviation: HR, Hazard Ratio; CI: Confidence Interval; *,P<0.05 | | | | | | |
